# A New Cell Topology for 4H-SiC Planar Power MOSFETs for High-Frequency Switching

**DOI:** 10.3390/ma15196690

**Published:** 2022-09-27

**Authors:** Shengnan Zhu, Tianshi Liu, Junchong Fan, Arash Salemi, Marvin H. White, David Sheridan, Anant K. Agarwal

**Affiliations:** 1 Department of Electrical and Computer Engineering, The Ohio State University, Columbus, OH 43210, USA; 2 Alpha and Omega Semiconductor, Sunnyvale, CA 94085, USA

**Keywords:** SiC power MOSFET, cell topology, dodecagonal cell, octagonal cell, gate-to-drain capacitance (C_gd_), specific ON-resistance (R_on_,_sp_), high-frequency figure of merit (HF-FOM), switching performance

## Abstract

A new cell topology named the dodecagonal (a polygon with twelve sides, short for Dod) cell is proposed to optimize the gate-to-drain capacitance ($C_{\mathrm{gd}}$) and reduce the specific ON-resistance ($R_{\mathrm{on},\mathrm{sp}}$) of 4H-SiC planar power MOSFETs. The Dod and the octagonal (Oct) cells are used in the layout design of the 650 V SiC MOSFETs in this work. The experimental results confirm that the Dod-cell MOSFET achieves a 2.2× lower $R_{\mathrm{on},\mathrm{sp}}$, 2.1× smaller high-frequency figure of merit (HF-FOM), higher turn on/off dv/dt, and 29% less switching loss than the fabricated Oct-cell MOSFET. The results demonstrate that the Dod cell is an attractive candidate for high-frequency power applications.

## 1. Introduction

The silicon carbide (SiC) power MOSFET has been expected to be the alternative to silicon power devices in multiple applications, such as renewable energy, drivers for electrical machines, and power converters for hybrid and electric vehicles, due to its various advantages, including a low power loss, high switching frequency, and high operating temperature [1,2,3]. Improving energy efficiency (reducing power dissipation) is the driving force for the commercialization of SiC power MOSFETs [3]. The total power loss of a SiC power MOSFET consists of an on-state conduction loss and switching loss. The conduction loss can be reduced by reducing the ON-resistance ($R_{\mathrm{on}}$). The switching loss of a SiC power MOSFET can be reduced by decreasing device capacitances [4,5]. Studies show that the layout topology design affects the on-state and dynamic performances of SiC power devices [6,7]. Different cell topologies (Linear, Hexagonal, Square, and Octagonal) were used on 600 V SiC planar MOSFETs [7]. All the figure of merits (FOMs) for the Octagonal cell topology are comparable to those of the state-of-the-art Si COOLMOS and are much better than the ROHM SiC MOSFET product [7]. In addition, simulations show that the oxide electric field for an Octagonal cell is much lower in comparison to other topologies [7]. The Linear, Hexagonal, and Octagonal cell topologies were used on 650 V SiC planar JBSFETs and reported in [8]. It is demonstrated that the Hexagonal cell topology provides the lowest specific ON-resistance ($R_{\mathrm{on},\mathrm{sp}}$) but a higher gate–drain charge than Linear and Octagonal cells [8]. It indicates that the Hexagonal cell is suitable for power-switching applications at a nominal frequency. However, for applications where a faster switching frequency is desired, such as reducing the size of power converters, the layout, such as the Octagonal (Oct) cell, is preferred, which tends to reduce the $C_{\mathrm{gd}}$ at the cost of a higher $R_{\mathrm{on}}$ [9].

In this work, a new cell topology named the dodecagonal (Dod) cell is proposed and compared with the Oct cell. Additionally, 650 V SiC power MOSFETs with the Dod and Oct cells have been designed, fabricated, and characterized. The static and dynamic performances of the fabricated 650 V SiC power MOSFETs are compared.

## 2. Device Design and Fabrication

**Figure 1:**
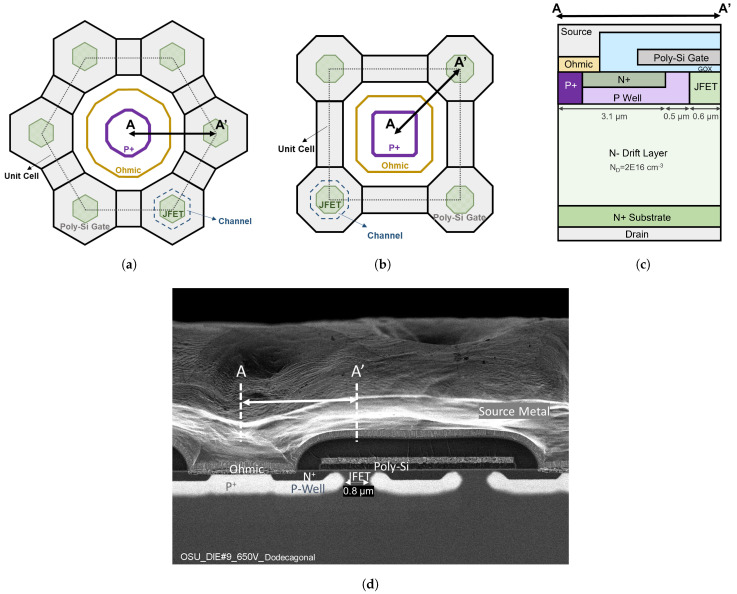
(**a**) Dodecagonal cell topology, (**b**) Octagonal cell topology, (**c**) A-A’ cross-sectional view of the 650 V SiC power MOSFET. (**d**) Cross-sectional SEM image of the fabricated 650 V SiC power MOSFET with the Dod cell.

Figure 1a shows the structure of the proposed Dod cell. A twelve-sided $P^{+}$ region with the ohmic contact on top is surrounded by six hexagonal poly-Si gate regions. The hexagonal gate regions are connected by poly-Si bars. The hexagonal JFET regions are placed inside gate regions. This Dod cell is applied to 650 V SiC power MOSFETs. The Oct cell is shown in Figure 1b. The cross-sectional views along the AA’ direction for both Dod-cell and Oct-cell MOSFETs in this work are shown in Figure 1c. All MOSFETs have the same edge termination design and die size. The die size is 1.15 × 1.15 ${\mathrm{mm}}^{2}$, including the termination. The MOSFETs are fabricated on a 6-inch SiC wafer by X-Fab using the same SiC power MOSFET process. Figure 1d shows the cross-sectional SEM image of the fabricated Dod-cell MOSFETs. Due to the lateral straggle of Aluminum implantation in the P-well, the narrowest portion of the JFET region is reduced by 0.2 μm on each side. Five Dod-cell and five Oct-cell MOSFETs are packaged into open-cavity TO-247 packages for device characterization. Design parameters and experimental results are listed in Table 1.

## 3. Experimental Results and Discussion

### 3.1. Statistic Performance

**Figure 2:**
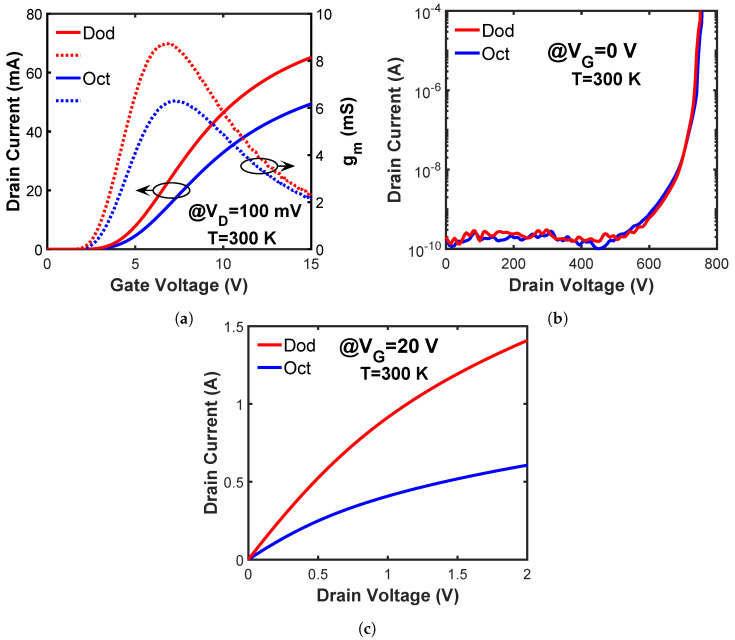
(**a**) Transfer characteristics at $V_{D}$ = 100 mV (solid line: drain current vs. gate voltage; dash line: transconductance vs. gate voltage), (**b**) blocking characteristics at $V_{G}$ = 0 V, and (**c**) output characteristics at $V_{G}$ = 20 V, for the 650 V SiC power MOSFETs with Dod and Oct cells.

The static performance, including the transfer, blocking, and output characteristics, is measured using a Keysight 1506A power semiconductor analyzer at room temperature. The results of one MOSFET from each cell type are shown in Figure 2. The threshold voltages ($V_{\mathrm{th}}$) of all packaged MOSFETs are extracted using the linear extrapolation method from the transfer curves, as shown in Figure 2a. The average value and the standard derivation are shown in Table 1. A minimal $V_{\mathrm{th}}$ difference (< 0.3 V) is observed between the MOSFETs with Dod and Oct cells. The transconductances ($g_{m}$) are also plotted in Figure 2a. The peak $g_{m}$ value for the Dod-cell MOSFET is 1.5× higher than the fabricated Oct-cell MOSFET, indicating a higher current-driving capability. The blocking characteristics (Figure 2b) show less than 1 nA leakage current up to 600 V for both the Dod-cell and Oct-cell MOSFETs. The breakdown voltage (BV) is defined at the drain current of 100 μA. Similar average BVs and BV standard derivations are obtained for the Oct-cell and Dod-cell MOSFETs, as shown in Table 1. Therefore, the Dod cell does not degrade the blocking capability of the fabricated power MOSFET.

The output characteristics are shown in Figure 2c. A 2× higher drain current is observed on the fabricated Dod-cell MOSFET than on the Oct-cell MOSFET, which can be explained by the higher channel density and JFET density of the Dod cell. A higher channel density (total channel width in a unit cell/unit cell area) implies a larger total channel width and more current conduction within a specific die size. The channel densities for the Dod and Oct cells are calculated according to the geometry with the known cross section and cell pitch. The results are listed in Table 1. A 1.6× higher channel density is obtained for the Dod cell than the Oct cell. The higher channel density allows more current conduction for the fabricated Dod-cell MOSFET, resulting in a lower $R_{\mathrm{on},\mathrm{sp}}$ than the fabricated Oct-cell MOSFET. The higher JFET density (JFET area/unit cell area) indicates a lower JFET region resistance, which contributes to the lower $R_{\mathrm{on},\mathrm{sp}}$ [10]. The $R_{\mathrm{on},\mathrm{sp}}$ of all the measured MOSFETs is calculated at a drain–source voltage ($V_{D}$) of 1.5 V. The mean values and standard derivations are listed in Table 1. A 2.2× reduction in the $R_{\mathrm{on},\mathrm{sp}}$ is achieved for the Dod-cell MOSFET compared to the fabricated Oct-cell MOSFET.

### 3.2. Dynamic Performance

**Figure 3:**
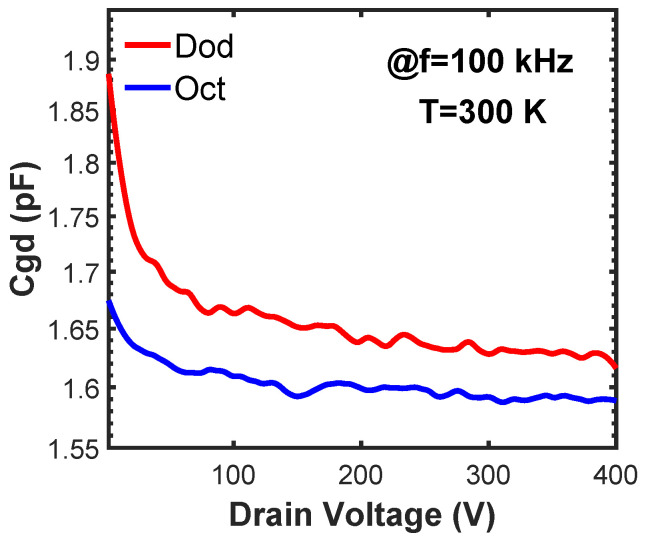
The $C_{\mathrm{gd}}$ of 650 V SiC MOSFETs with the Dod and the Oct cells.

The $C_{\mathrm{gd}}$ vs. drain voltage characteristics are shown in Figure 3. The $C_{\mathrm{gd}}$ significantly affects the dynamic behavior of SiC MOSFETs due to the well-known Miller effect [11,12]. The $C_{\mathrm{gd}}$ of a SiC planar MOSFET consists of the gate oxide capacitance on the top of the JFET region and the depletion capacitance of the JFET region and drift layer [13]. The results in Figure 3 show that the Dod-cell MOSFET has a higher $C_{\mathrm{gd}}$ than the fabricated Oct-cell MOSFET under any drain bias. Because the Dod-cell and Oct-cell MOSFETs are fabricated following the same process flow, their gate oxide thickness, JFET doping profile, and drift-layer doping profile are the same. Thus, the difference in the $C_{\mathrm{gd}}$ is caused by the different overlap areas of the gate and drain terminals (JFET region area) within a die. The calculated JFET density of the Dod cell is 1.6× higher than the Oct cell, which causes a higher $C_{\mathrm{gd}}$ of the Dod-cell MOSFET. The $C_{\mathrm{gd}}$ values at $V_{D}$ = 400 V are extracted for all the measured MOSFETs. Additionally, the HF-FOMs, which are defined as the product of $R_{\mathrm{on}}$ and $C_{\mathrm{gd}}$, are calculated. The HF-FOM of the Dod-cell MOSFET is 2.1× smaller than the fabricated Oct-cell MOSFET, indicating a better high-frequency performance.

**Figure 4:**
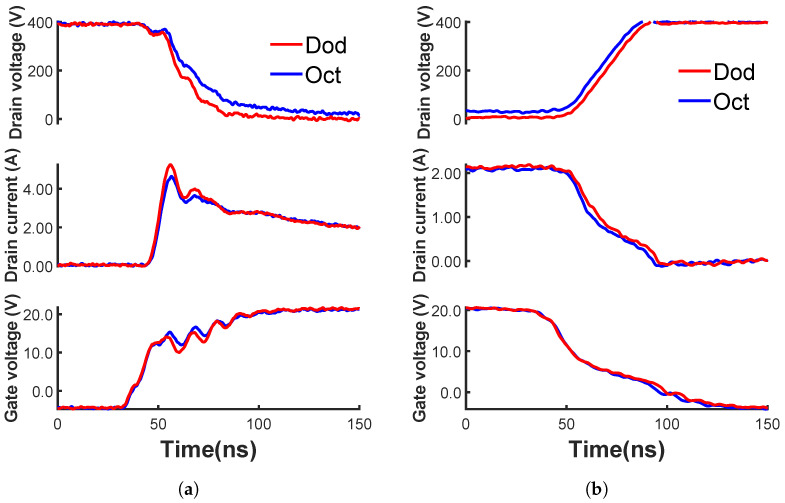
(**a**) Turn-on and (**b**) turn-off waveforms of the fabricated 650 V SiC power MOSFETs with the Dod and the Oct cells.

A Double-Pulse Test (DPT), which provides straightforward data on the switching behavior of SiC MOSFETs [14], is conducted to investigate the switching performance of the fabricated MOSFETs. The results are shown in Figure 4. During the turn-on process (Figure 4a), the Dod-cell MOSFET shows a shorter transient time (higher turn-on speed) than the fabricated Oct-cell MOSFET. The turn-on and turn-off dv/dt, switching energy, and total switching energy of the measured MOSFETs are extracted and listed in Table 1. The Dod-cell MOSFET has a higher dv/dt and less switching energy than the measured Oct-cell MOSFET during both the turn-on and turn-off processes. The Dod-cell MOSFET obtains a 29% reduction in total switching loss compared to the measured Oct-cell MOSFET.

## 4. Conclusions

A new cell topology named the Dod cell is proposed for SiC planar power MOSFETs. The Dod cell minimizes the JFET region area to achieve a low $C_{\mathrm{gd}}$ and improves the channel density to reduce the $R_{\mathrm{on},\mathrm{sp}}$. A low $C_{\mathrm{gd}}$ indicates a higher switching speed and makes the Dod-cell MOSFET an attractive candidate for high-frequency applications. The reduced $R_{\mathrm{on},\mathrm{sp}}$ decreases the conduction loss. The Dod cell and a recently published Oct cell (designed with minimal JFET region area) have been used for the layout design of 650 V SiC power MOSFETs. The fabricated Dod-cell MOSFET achieves a higher $C_{\mathrm{gd}}$, but a 2.2× lower $R_{\mathrm{on},\mathrm{sp}}$, 2.1× smaller HF-FOM, and 29% less switching losses compared with the fabricated Oct-cell MOSFET.

## Figures and Tables

**Table 1:** Design parameters and experimental results.

Cell Topology		Oct	Dod
Design parameters	Cell pitch (μm)	8.4	8.4
	Active area (mm${}^{2}$)	0.643	0.634
	Channel density (μm${}^{-1}$)	0.113	0.181
	JFET density	0.034	0.055
Experimental results	$V_{\mathrm{th}}$ (V)	4.09 ± 0.09 *	4.35 ± 0.10
	BV (V) @ $I_{D}$ = 100 μA	756.3 ± 22.6	753.4 ± 23.6
	$R_{\mathrm{on},\mathrm{sp}}$ (mΩ·cm${}^{2}$) @ $V_{D}$ = 1.5 V	24.4 ± 7.52	10.9 ± 3.06
	$C_{\mathrm{gd}}$ (pF) @ $V_{D}$ = 400 V	1.59 ± 0.01	1.63 ± 0.04
	HF-FOM (mΩ·pF) ($R_{\mathrm{on}}\;\cdot \;C_{\mathrm{gd}}$)	6031 ± 1896	2817 ± 749
	dv/dt turn-on (V/ns)	6.1	12.5
	Switching loss turn-on (μJ)	29.4	20.1
	dv/dt turn-off (V/ns)	8.4	10.7
	Switching loss turn-off (μJ)	5.7	4.7
	Total switching loss (μJ)	35.1	24.8

* ± refers to the standard deviation of the value.

## Data Availability

Not applicable.

## Abbreviations

The following abbreviations are used in this manuscript:

SiC	Silicon Carbide
MOSFET	Metal-Oxide-Semiconductor Field-Effect Transistor
JFET	Junction Field-Effect Transistor
JBSFET	Junction Barrier Schottky (JBS) diode-integrated MOSFET
HF-FOM	High-Frequency Figure of Merit
DPT	Double-Pulse Test

## References

[1] Camacho A.P., Sala V., Ghorbani H., Martinez J.L.R. (2017). A novel active gate driver for improving SiC MOSFET switching trajectory. IEEE Trans. Ind. Electron..

[2] Hazra S., De A., Cheng L., Palmour J., Schupbach M., Hull B.A., Allen S., Bhattacharya S. (2015). High switching performance of 1700-V, 50-A SiC power MOSFET over Si IGBT/BiMOSFET for advanced power conversion applications. IEEE Trans. Power Electron..

[3] Kimoto T. (2015). Material science and device physics in SiC technology for high-voltage power devices. Jpn. J. Appl. Phys..

[4] Baliga B.J. (1989). Power semiconductor device figure of merit for high-frequency applications. IEEE Electron Device Lett..

[5] Wang H., Wang F., Zhang J. (2007). Power semiconductor device figure of merit for high-power-density converter design applications. IEEE Trans. Electron Devices.

[6] Watanabe N., Okino H., Shima A. Impact of Cell Layout on On-state and Dynamic Characteristics of N-channel SiC IGBTs. Proceedings of the 2022 IEEE 34th International Symposium on Power Semiconductor Devices and ICs (ISPSD).

[7] Agarwal A., Han K., Baliga B.J. (2019). Impact of cell topology on characteristics of 600V 4H-SiC planar MOSFETs. IEEE Electron Device Lett..

[8] Agarwal A., Han K., Baliga B. (2020). Assessment of linear, hexagonal, and octagonal cell topologies for 650 V 4H-SiC inversion-channel planar-gate power JBSFETs fabricated with 27 nm gate oxide thickness. IEEE J. Electron Devices Soc..

[9] Han K., Baliga B. (2018). The 1.2-kV 4H-SiC OCTFET: A new cell topology with improved high-frequency figures-of-merit. IEEE Electron Device Lett..

[10] Liu T., Zhu S., Salemi A., Sheridan D., White M.H., Agarwal A.K. (2021). JFET Region Design Trade-Offs of 650 V 4H-SiC Planar Power MOSFETs. Solid State Electron. Lett..

[11] Mukunoki Y., Nakamura Y., Konno K., Horiguchi T., Nakayama Y., Nishizawa A., Kuzumoto M., Akagi H. (2018). Modeling of a silicon-carbide MOSFET with focus on internal stray capacitances and inductances, and its verification. IEEE Trans. Ind. Appl..

[12] Ebihara Y., Ichimura A., Mitani S., Noborio M., Takeuchi Y., Mizuno S., Yamamoto T., Tsuruta K. Deep-P encapsulated 4H-SiC trench MOSFETs with ultra low R on Q gd. Proceedings of the 2018 IEEE 30th International Symposium on Power Semiconductor Devices and ICs (ISPSD).

[13] Zhu S., Liu T., Fan J., Maddi H.L.R., White M.H., Agarwal A.K. (2022). Effects of JFET Region Design and Gate Oxide Thickness on the Static and Dynamic Performance of 650 V SiC Planar Power MOSFETs. Materials.

[14] Li X., Jiang J., Huang A.Q., Guo S., Deng X., Zhang B., She X. (2017). A SiC power MOSFET loss model suitable for high-frequency applications. IEEE Trans. Ind. Electron..

